# Supplementary material for: Feasibility of Brewer’s Spent Yeast Microcapsules as Targeted Oral Carriers
Source: Foods. 2023 Jan 5;12(2):246. doi: 10.3390/foods12020246 (PMC9857821; doi:10.3390/foods12020246)
Supplement: Supplementary file 1 [file foods-12-00246-s001.zip › Supplementary Table S1. List of polysaccharides in the array .pdf]

Supplementary Table S1 - List of polysaccharide probes included in the microarray and their main composition.

Position[a]	Probe designation[b]	Predominant oligosaccharide sequence or monosaccharide composition[c]
BSY		
1	*1M KOH*	Carbohydrates (35%): Glc (58%), Man (42%)<br>4-Glc (29%), 6-Glc (2%), 3-Glc (0.2%)
2	*4M KOH*	Carbohydrates (44%): Man (67%), Glc (33%)<br>4-Glc (22%), 6-Glc (3%), 3-Glc (0.3%)
3	*180 SWE*	Carbohydrates (33%): Man (68%), Glc (32%)<br>3-Glc (16%), 4-Glc (5%), 6-Glc (2%)
4	*200 SWE*	Carbohydrates (23%): Man (54%), Glc (46%)<br>3-Glc (27%), 4-Glc (7%), 6-Glc (2%)
Glucans		
5	NSG-β-glucan	Linear β1,3-Glc backbone with occasional monoglucosyl β1,6-Glc branches
6	Lichenan	Mixed-linked β1,3/1,4-Glc; 1:2 linkage ratio
7	Barley	Mixed-linked β1,3/1,4-Glc; 1:3-4 linkage ratio; contains Ara (2%), Xyl (0.2%)
8	Oat	Mixed-linked β1,3/1,4-Glc
9	PGG-β-glucan	Linear β1,3-Glc backbone with occasional monoglucosyl β1,6-Glc branches
Mannans		
10	Mannoprotein (MP1)	Yeast cell wall; sequential extraction 4M KOH<br>Ara (1%), Xyl (1%), Man (85%), Glc (13%)
11	Mannoprotein (MP2)	Yeast cell wall; sequential extraction 8M KOH<br>Ara (1%), Xyl (0%), Man (65%), Glc (35%)
12	Mannan (*S. cerevisae*)	α1,6-Man backbone with oligomeric α1,2-, α1,3-Man branches

[a]Position of the polysaccharide probes in the charts.

[b]Yeast fractions (#1-4) are solubilized material after *in vitro* digestion of brewer's spent yeast microcapsules resultant from alkaline extractions (1M KOH and 4M KOH) and subcritical water extractions (SWE) using microwave at different temperatures (180 SWE and 200 SWE). The source of glucans are as follows: NSG-β-glucan (#5) from *S. cerevisiae*, Biothera; Lichenan (#6) from Icelandic moss, Megazyme (P-LICHN); Barley (#7) from barley flour, Megazyme (P-BGBL); Oat (#8) from oat flour, Megazyme (P-BGOM), PGG from *S. cerevisiae*, Biothera. These were previously described (Palma *et al.*, 2015). Mannoproteins MP1 (#10) and MP2 (#11) were isolated from yeast cell walls and were previously described (Pinto, Coelho, Nunes, Brandão, & Coimbra, 2015); Mannan from *S. cerevisae* (#12) was from Sigma.

[c]For the BSYIM fractions and mannoproteins MP1 and MP2, the main sugars (mol %) and glycosidic linkages (relative molar ratio, %) identified by sugar and methylation analysis are shown.

Abbreviations: Ara, arabinose; BSYIM, brewer's spent yeast insoluble material; Glc, glucose, Man, mannose, NSG, neutral soluble glucan; PGG, poly-(1,6)-D-glucopyranosyl-(1,3)-D-glucopyranose; Xyl, xylose.

Supplementary References:

Palma, A. S., Liu, Y., Zhang, H., Zhang, Y., McCleary, B. V., Yu, G., Huang, Q., Guidolin, L. S., Ciocchini, A. E., Torosantucci, A., Wang, D., Carvalho, A. L., Fontes, C. M., Mulloy, B., Childs, R. A., Feizi, T., & Chai, W. (2015). Unravelling glucan recognition systems by glycome microarrays using the designer approach and mass spectrometry. *Mol Cell Proteomics,* 14(4), 974-988.

Pinto, M., Coelho, E., Nunes, A., Brandão, T., & Coimbra, M. A. (2015). Valuation of brewers spent yeast polysaccharides: a structural characterization approach. *Carbohydr Polym,* 116, 215-222.
