# Supplementary material for: Feasibility of Brewer’s Spent Yeast Microcapsules as Targeted Oral Carriers
Source: Foods. 2023 Jan 5;12(2):246. doi: 10.3390/foods12020246 (PMC9857821; doi:10.3390/foods12020246)
Supplement: Supplementary file 1 [file foods-12-00246-s001.zip › Supplementary Table S2. List of proteins analysed in the array .pdf]

Supplementary Table S2. List of proteins used for quality control of microarray, their sources, conditions of analysis and their reported carbohydrate-binding specificity.

Proteins	Source[a]	Reported carbohydrate-binding specificity / Recognition[b]	Analysis conc.	Detection reagents & concentrations[c]	Blocking solution[d]
**LECTINS**					
**Human Dectin-1**	Sino Biological (10215-H01H)	β1,3-glucooligomers with a minimum chain length of DP-10 or DP-11 (Palma *et al.*, 2006; Palma *et al.*, 2015)	10 µg/ml	BI anti-human IgG (Vector BA-3000); 10 µg/mL	Blocker A
**Human DC-SIGN** (Dendritic cell-specific ICAM-3-grabbing non-integrin)	Sino Biological (10200-H01H)	Recognition of fuco- (Silva *et al.*, 2021), manno- (Gao *et al.*, 2021; Geissner *et al.*, 2019; Vendele *et al.*, 2020) and gluco-oligosaccharides (Palma *et al.*, 2015)	10 µg/ml	BI anti-human IgG (Vector BA-3000); 10 µg/mL	Blocker A
**Mouse Dectin-2**	R&D Systems (1525-DC-050)	Recognition of α-mannose residues, such as high mannose-type *N*-glycans ($Man_8GlcNAc_2$) (Vendele *et al.*, 2020)	5 µg/mL	Ab1: mouse monoclonal anti-poly-Histidine (Sigma SAB4200620); 15 µg/mL; Ab2: BI anti-mouse IgG (whole molecule) (Sigma B7264); 15 µg/mL	Blocker A
**ConA** (Concanavalin A)	Vector (B-1005-5)	Terminal α-Man-linked oligosaccharides (Pejchal *et al.*, 2011)<br>Terminal α- or β-Glc-linked oligosaccharides (Geissner *et al.*, 2019; Palma *et al.*, 2015)	5 µg/mL	N/A	Blocker B
**MONOCLONAL ANTIBODIES**					
**400-2**	Biosupplies	Raised against β1,3-glucan laminarin;<br>β1,3-glucose oligosaccharide sequences in β1,3-glucans (DP ≥ 2) (P. J. Meikle, Bonig, Hoogenraad, Clarke, & Stone, 1991).	15 µg/mL	BI anti-mouse IgG (Sigma B7264); 10 µg/mL	Blocker A

[a]The commercial source is indicated.

[b]The main carbohydrate-binding specificity assigned is indicated with a reference.

[c]Ab1, primary antibody; Ab2, secondary antibody; BI, biotinylated; N/A, not applicable.

[d]Blocker A: 1% (w/v) BSA, 0.02% (v/v) casein in HBS, 5 mM $CaCl_2$; Blocker B: 3% (w/v) BSA in HBS, 5 mM $CaCl_2$. HBS, 10 mM HEPES-buffered saline (pH 7.4), 150 mM NaCl ; BSA, bovine serum albumin (Sigma A8577); Casein (Pierce 37528).

Supplementary References:

Gao, C., Stavenhagen, K., Eckmair, B., McKitrick, T. R., Mehta, A. Y., Matsumoto, Y., McQuillan, A. M., Hanes, M. S., Eris, D., Baker, K. J., Jia, N., Wei, M., Heimburg-Molinaro, J., Ernst, B., & Cummings, R. D. (2021). Differential recognition of oligomannose isomers by glycan-binding proteins involved in innate and adaptive immunity. *Sci Adv,* 7(24).

Geissner, A., Reinhardt, A., Rademacher, C., Johannssen, T., Monteiro, J., Lepenies, B., Thepaut, M., Fieschi, F., Mrazkova, J., Wimmerova, M., Schuhmacher, F., Gotze, S., Grunstein, D., Guo, X., Hahm, H. S., Kandasamy, J., Leonori, D., Martin, C. E., Parameswarappa, S. G., Pasari, S., Schlegel, M. K., Tanaka, H., Xiao, G., Yang, Y., Pereira, C. L., Anish, C., & Seeberger, P. H. (2019). Microbe-focused glycan array screening platform. *Proceedings of the National Academy of Sciences of the United States of America,* 116(6), 1958-1967.

Meikle, P. J., Bonig, I., Hoogenraad, N. J., Clarke, A. E., & Stone, B. A. (1991). The location of (1→3)-β-glucans in the walls of pollen tubes of Nicotiana alata using a (1→3)-β-glucan-specific monoclonal antibody. *Planta,* 185(1), 1-8.

Palma, A. S., Feizi, T., Zhang, Y., Stoll, M. S., Lawson, A. M., Díaz-Rodríguez, E., Campanero-Rhodes, M. A., Costa, J., Gordon, S., Brown, G. D., & Chai, W. (2006). Ligands for the β-Glucan Receptor, Dectin-1, Assigned Using "Designer" Microarrays of Oligosaccharide Probes (Neoglycolipids) Generated from Glucan Polysaccharides*. *Journal of Biological Chemistry,* 281(9), 5771-5779.

Palma, A. S., Liu, Y., Zhang, H., Zhang, Y., McCleary, B. V., Yu, G., Huang, Q., Guidolin, L. S., Ciocchini, A. E., Torosantucci, A., Wang, D., Carvalho, A. L., Fontes, C. M., Mulloy, B., Childs, R. A., Feizi, T., & Chai, W. (2015). Unravelling glucan recognition systems by glycome microarrays using the designer approach and mass spectrometry. *Mol Cell Proteomics,* 14(4), 974-988.

Pejchal, R., Doores, K. J., Walker, L. M., Khayat, R., Huang, P. S., Wang, S. K., Stanfield, R. L., Julien, J. P., Ramos, A., Crispin, M., Depetris, R., Katpally, U., Marozsan, A., Cupo, A., Maloveste, S., Liu, Y., McBride, R., Ito, Y., Sanders, R. W., Ogohara, C., Paulson, J. C., Feizi, T., Scanlan, C. N., Wong, C. H., Moore, J. P., Olson, W. C., Ward, A. B., Poignard, P., Schief, W. R., Burton, D. R., & Wilson, I. A. (2011). A Potent and Broad Neutralizing Antibody Recognizes and Penetrates the HIV Glycan Shield. *Science,* 334(6059), 1097-1103.

Silva, L. M., Correia, V. G., Moreira, A. S. P., Domingues, M. R. M., Ferreira, R. M., Figueiredo, C., Azevedo, N. F., Marcos-Pinto, R., Carneiro, F., Magalhães, A., Reis, C. A., Feizi, T., Ferreira, J. A., Coimbra, M. A., & Palma, A. S. (2021). Helicobacter pylori lipopolysaccharide structural domains and their recognition by immune proteins revealed with carbohydrate microarrays. *Carbohydr Polym,* 253, 117350.

Vendele, I., Willment, J. A., Silva, L. M., Palma, A. S., Chai, W., Liu, Y., Feizi, T., Spyrou, M., Stappers, M. H. T., Brown, G. D., & Gow, N. A. R. (2020). Mannan detecting C-type lectin receptor probes recognise immune epitopes with diverse chemical, spatial and phylogenetic heterogeneity in fungal cell walls. *PLoS Pathog,* 16(1), e1007927.
